# The importance of iron in pathophysiologic conditions

**DOI:** 10.3389/fphar.2015.00026

**Published:** 2015-02-24

**Authors:** Raffaella Gozzelino, Paolo Arosio

**Affiliations:** ^1^Inflammation Laboratory, Instituto Gulbenkian de CiênciaOeiras, Portugal; ^2^Department of Molecular and Translational Medicine, University of BresciaBrescia, Italy

**Keywords:** iron, iron metabolism, iron and genetic disorders, iron deficiency and anemia, iron and inflammation, iron and cardiotoxicity, iron and neurodegeneration, heme iron

Biological iron is necessary for vital functions and also potentially toxic to the organisms. This dual effect raised the interest of many investigators to study the mechanisms controlling its homeostasis that are altered in many pathologic conditions. Recently the understanding of iron metabolism significantly improved with the discovery of genes responsible for genetic disorders, such as hemochromatosis, the IRE/IRPs machinery and the hepcidin-ferroportin axis, which allowed to elucidate the basis of cellular and systemic iron homeostasis. In addition, these advances disclosed a causal link between deregulation of iron homeostasis, inflammation and oxidative stress, often induced by the iron accumulation that is commonly observed in many pathologic conditions.

Hence, believing this was time to provide a current state-of-the-art on the importance of iron in pathophysiologic conditions, we thought to promote a Research Topic with the contribution of top-leading scientists who studied the effects of iron homeostasis disruption on the outcome of genetic, inflammatory, infectious, cardiovascular, and neurodegenerative diseases. Encountering an interest even larger than our ambitions, we successfully collected 42 manuscripts, which cover the major aspects of iron metabolism, from the essential role of iron for cell survival to its contribution in the pathogenesis of various disorders. They have been organized in an e-book in 7 sections.

The first section of the Research Topic includes 11 papers that cover the importance of iron in cellular proliferation, differentiation and functioning, and its crucial role in essential processes such as oxygen transport, DNA synthesis, metabolic energy and cellular respiration. They describe the expression and regulation of the main players involved in the mechanisms of iron absorption, recycling, and mobilization (Zhang et al., 2014), the cooperation among different cellular compartments that facilitates iron mobilization/storage and prevents the deleterious effects induced by its accumulation, the role of iron in the Fenton chemistry and its effects on oxidative stress and programmed cell death. Of interest is the study of the different types of circulating iron and the strategies more commonly used for its detection (Cabantchik, 2014). The papers also present updates on iron metabolism in zebrafish, in *C. elegans*, the role of iron in the skin and the regulatory mechanisms devoted to iron uptake, recycling and mobilization. Altogether they provide the information for a better understanding of the iron involvement in pathophysiologic conditions.

The second section includes three papers dealing with the role of heme inside cells and its cytotoxicity when it is released from hemoproteins. In fact, most body iron is contained within the protoporphyrin ring that acts as prosthetic group in many hemoproteins essential to cellular functions. The different aspects related to heme synthesis, intracellular trafficking, scavenging and catabolism are reviewed together with the protective mechanisms that cooperate to prevent the deleterious effects induced by heme accumulation and the pathological conditions in which heme plays a dominant role (Chiabrando et al., 2014). The expression and regulation of the main heme scavengers and transporters identified are also reviewed, together with the notion that maintenance of heme homeostasis is essential to prevent the deleterious effect induced by its overload (Korolnek and Hamza, 2014). The following sections are devoted to describe how disruption of iron homeostasis is associated with a series of syndromes and dictates the outcomes and severity of these disorders.

The third section deals with hemochromatosis, the genetic disease that has a fundamental importance for identifying the actors responsible for systemic iron regulation. It includes two reviews on the origin and genetic mutations characterizing this pathology as well as the incidence and different types of hemochromatosis identified so far (Vujic, 2014). The symptoms and manifestations that characterize these disorders and the alteration of the responsible proteins are also described (Silvestri et al., 2014).

The forth section deals with anemia and iron deficiency, problems that are common worldwide, and includes four papers. The distribution of iron deficiency in the population and in particular during aging are reviewed (Busti et al., 2014). The etiology of anemia, caused by genetic disorders, inflammation, infections, bleeding due to the development of ulcers, drug administration or cancers are covered. The roles of TMPRSS6 and of its substrate, hemojuvelin, in the regulation of BMP signaling and hepcidin expression are reviewed (Core et al., 2014; Wang et al., 2014). Finally, the increasing number of therapeutic approaches targeting the various steps involved in hepcidin regulation are summarized together with the promising results capable to correct altered hematological parameters in animal models (Poli et al., 2014).

The fifth section deals with one of the hottest topics of the field, which is the connection between iron and inflammation, largely mediated by hepcidin expression. Seven papers are included. One is an updated review on the known battle between host and pathogen for access to the iron necessary for proliferation, particular reference to the very complex malaria infection (Spottiswoode et al., 2014). The molecular mechanisms leading to disruption of iron homeostasis upon infections caused by parasites (Penha-Goncalves et al., 2014), intracellular or extracellular pathogens are also detailed (Nairz et al., 2014). Of importance are the effects of iron supplementation therapy to individuals suffering from infectious diseases (Clark et al., 2014) and the role of proteins that restricting iron availability to microbes may modify the outcome of the infection.

The sixth section discusses the role of iron overload in cardiovascular diseases, which includes six papers. One is a review on the long-standing and obscure relationship between iron availability and anthracycline cardiotoxicity, stressing the role of chelating agents and ferritins as agents protecting against the pro-oxidant activity of the drug (Gammella et al., 2014). A detailed overview on the pathways leading to the disruption of iron homeostasis and impairing heart functioning is described (Basuli et al., 2014). The toxicity of iron was addressed in different cell types, emphasizing in particular the effects exerted on macrophages for the development and progression of atherosclerotic plaque. In this section, a special attention is also given to the occurrence of cardiovascular abnormalities and death in hemochromatosis patients, thus further confirming the role of iron in the pathogenesis of these diseases (Vinchi et al., 2014).

Finally, the seventh section discusses the role of iron in neurodegenerative diseases and includes nine papers. Although the regulation of iron homeostasis in the brain remains rather obscure, its alteration has been observed in a variety of brain disorders, including Parkinson's, Alzheimer's, Huntington's, Prion and neurodegeneration with brain iron accumulation (NBIA). This stimulated many studies to verify if the local iron overload is one, or the main contributor to neuronal death (Wong and Duce, 2014). The pathogenic mechanisms leading to neurodegeneration that are associated to gene mutations of NBIAs are reviewed and they pose another tight connection between iron deregulation and oxidative damage (Levi and Finazzi, 2014), The involvement of inflammation in the establishment and progression of these pathologic conditions and its correlation to the disruption of iron homeostasis was also addressed (Urrutia et al., 2014). The beneficial effect of an oral iron chelator able to pass through the blood-brain barrier, the deferiprone, in scavenging excess iron from regional foci of siderosis is reviewed together with the ongoing clinical trials. The chelator is claimed to be able to relocate efficiently iron and to replenish iron-deprived regions, thus ameliorating the symptoms of iron maldistribution and suppressing the deleterious effects of its overload (Cabantchik et al., 2013).

In summary, this research topic enjoys the contribution of top-leading scientists aimed at providing a current state of the art on the importance of iron metabolism and its contribution in a variety of human disorders.

## Conflict of interest statement

The authors declare that the research was conducted in the absence of any commercial or financial relationships that could be construed as a potential conflict of interest.
